# Response to “Anticancer Drug Approval in China: From Acceleration of Access to Certainty of Benefits”

**DOI:** 10.34133/hds.0337

**Published:** 2025-10-28

**Authors:** Lixia Fu, Yimin Cui

**Affiliations:** ^1^ Institute of Clinical Pharmacology, Peking University First Hospital, Beijing, China.; ^2^ Department of Pharmacy Administration and Clinical Pharmacy, School of Pharmaceutical Sciences, Peking University, Beijing, China.; ^3^ Beijing Key Laboratory of Clinical Pharmacology and Translation of Innovative Drugs, Beijing, China.

Thank you for your thorough review and insightful comments on our research [1]. Your observations are highly pertinent and provide valuable insights for our continued examination of China’s evolving anticancer drug regulatory framework. As discussed in our paper, innovative drugs in China are defined as New Molecular Entities with initial indication approvals, while modified new drugs typically refer to new indications for existing drugs, which also include new dosages, formulations, or patient populations [2]. We believe the reasons why the innovative trials did not demonstrate shorter trial durations and experienced longer approval review times are multifactorial. One reason is that innovative drugs are novel compounds that have not been previously used, making their pivotal trials more complex. Additionally, regulatory authorities may exercise greater caution and require more time for review. However, these are speculative explanations that require verification in future studies.

We agree with your perspective that the rationale for “accelerated approval compromises” should be re-examined in the context of China’s rapidly advancing drug development capabilities. In recent years, China has achieved substantial breakthroughs in both the number of innovative drugs and their proportion within the global research and development landscape. This success can be largely attributed to China’s drug regulatory system reforms, particularly in the oncology field [3]. However, numerous international studies have highlighted that accelerated approval pathways implemented by the US Food and Drug Administration (FDA), European Medicines Agency, and China’s National Medical Products Administration, while providing patients with expedited access to novel anticancer therapies, simultaneously introduce regulatory uncertainties [4,5]. This uncertainty primarily stems from two sources: first, pivotal trials frequently employ single-arm trial (SAT) designs, and second, surrogate endpoints are widely used as efficacy assessment indicators [6]. However, these designs and indicators do not always translate into clear clinical benefits, particularly in terms of overall survival (OS) and quality of life (QOL) improvements. Furthermore, studies have shown that the FDA has withdrawn multiple accelerated approvals for anticancer drugs in recent years [7], which underscores the critical importance of scientifically rigorous trial designs in supporting drug benefit–risk assessments. Regarding the availability of OS and QOL data, this indeed reflects the inherent limitations of surrogate endpoint applications, which necessitates strengthened post-marketing surveillance to compensate for these shortcomings.

Notably, China’s Center for Drug Evaluation (CDE) has also recognized the regulatory uncertainties introduced by SAT designs and other related factors. Consequently, on 2023 March 14, the CDE issued the “Technical Guidance on the Applicability of Single-Arm Clinical Trials to Support Marketing Applications for Anticancer Drugs” [8], which clearly defines the applicable conditions and standards for SATs in supporting drug marketing applications, while maintaining that randomized controlled trials remain the “gold standard” for demonstrating drug efficacy. This guidance aims to scientifically guide the industry in correctly understanding and strictly applying SAT, while emphasizing the importance of post-marketing surveillance to ensure that therapeutic benefits outweigh the risks introduced by the inherent uncertainties of the trials.

Based on your recommendations and our research findings, we suggest that China’s regulatory system align with international regulatory practices by further improving post-marketing surveillance, strengthening international cooperation, and timely reviewing and withdrawing anticancer drugs that fail to provide clear clinical benefit evidence. Additionally, conducting more regulatory science research and implementing other measures could enhance the accessibility and affordability of anticancer drugs. We look forward to further academic exchanges with colleagues in the field on these important issues, working together to promote the scientific development of anticancer drug regulatory policies.
